# Supplementary material for: Comprehensive investigation of temporal and autism-associated cell type composition-dependent and independent gene expression changes in human brains
Source: Sci Rep. 2017 Jun 23;7:4121. doi: 10.1038/s41598-017-04356-7 (PMC5482876; doi:10.1038/s41598-017-04356-7)
Supplement: Supplementary file 1 — Supplementary information [file 41598_2017_4356_MOESM1_ESM.pdf]

Supplementary materials

Comprehensive investigation of temporal and autism-associated cell type composition-dependent and independent gene expression changes in human brains

Qianhui Yu[1,2], Zhisong He[1*]

[1] CAS Key Laboratory of Computational Biology, CAS-MPG Partner Institute for Computational Biology (PICB), Shanghai Institutes for Biological Sciences (SIBS), Chinese Academy of Sciences (CAS), Shanghai, China

[2] University of Chinese Academy of Sciences, Beijing, China

Correspondence:

Zhisong He

zhisong.he@gmail.com

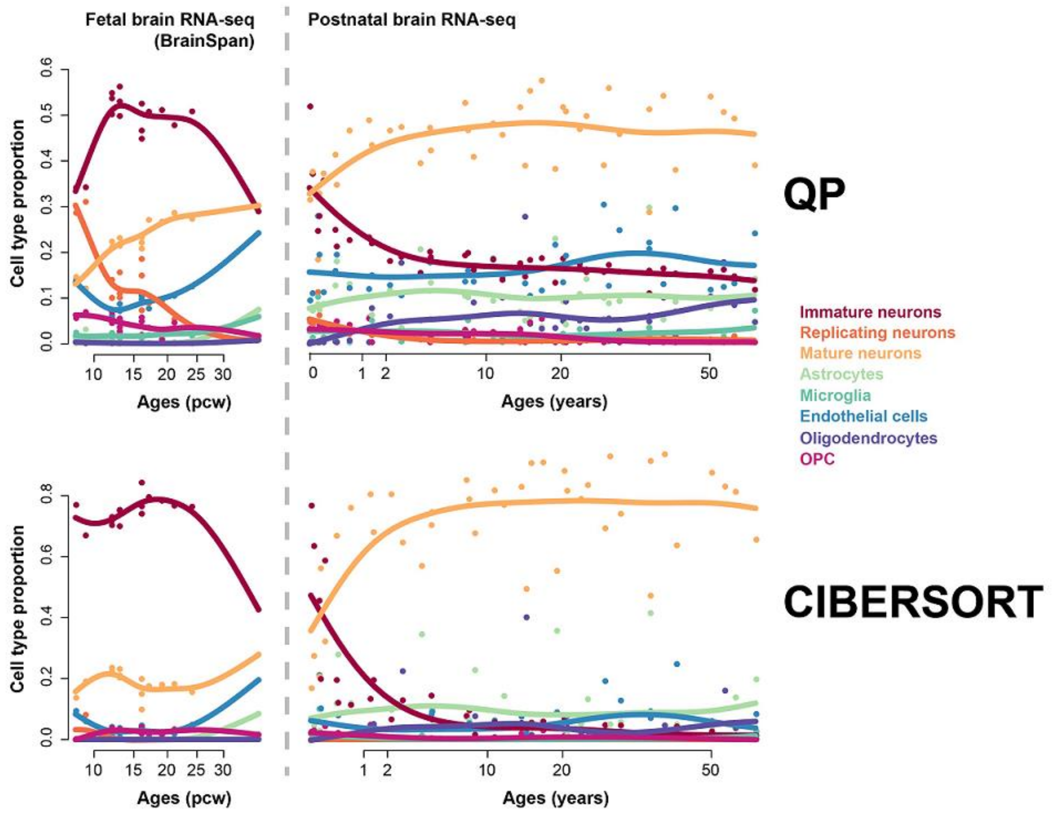

Supplementary Figure 1. The cell type compositions across lifespan in human brains, using QP (upper) and CIBERSORT (lower) algorithms. The dots show the estimated proportions of samples, and the curves show the spline interpolation results. The left panel shows the cell type compositions in human fetal brains, based on the human embryonic developmental brain RNA-seq data from BrainSpan; the right panel shows the cell type compositions in human postnatal brains, based on the human postnatal brain data set.

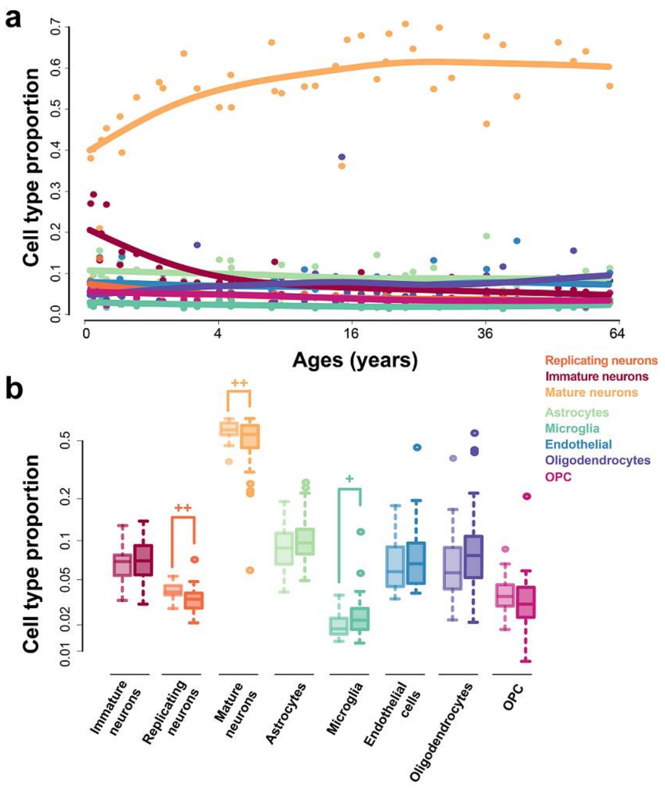

Supplementary Figure 2. Cell type compositions in human healthy and autistic brains, estimated using the DR-based deconvolution with confounding factor sensitive genes excluded. (a) The cell type compositions across lifespan in human brains. The dots show the estimated proportions of samples, and the curves show the spline interpolation results. (b) The estimated proportions of each cell types in ASD case samples and healthy control samples with ages at the same age range. Each pair of boxes with the same color show the proportions of one cell type, with the left box represents the proportions in healthy samples, and the right box represents the proportions in ASD samples. The plus signs mark significant difference according to Wilcoxon's test (+ – P<0.1; ++ – P<0.05).

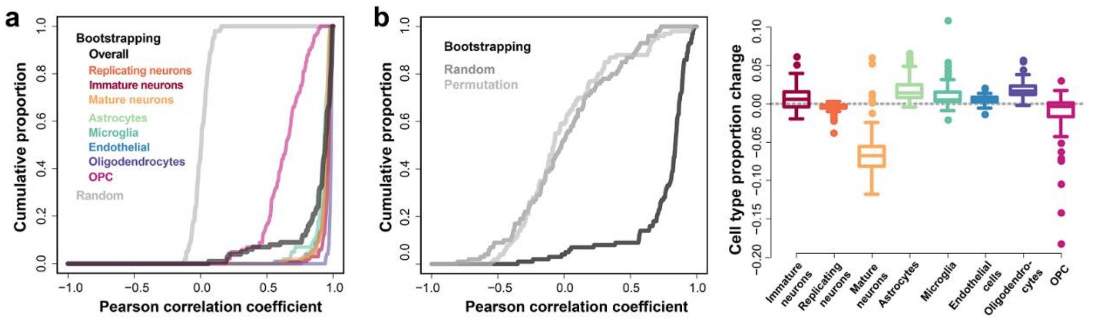

Supplementary Figure 3. Robustness of brain cell type composition temporal pattern and autistic brain composition alterations to individual cell variability. (a) The estimated cell type composition pattern is robust to cell variability of the single cell RNA-seq data. The curves show accumulative frequency distribution of Pearson correlation coefficient (PCC) between the reported pattern and patterns based on 100 bootstrapping of cells in Darmanis dataset. The black curve shows the robustness of the overall pattern, and curves with different colors represent the eight main cell types. The grey curve shows the correlations to random composition patterns. (b) The estimated cell type composition alterations in autism are robust to cell variability of the single cell RNA-seq data. In the left panel, the black curve shows accumulative frequency distribution of PCC between the average composition changes of the eight cell types based on the complete Darmanis dataset and changes estimated based on 100 bootstrapping of cells in Darmanis dataset. The darker grey curve shows PCC to normal distributed random changes. The lighter grey curve shows PCC to changes based on 100 permutation of cell types. In the right panel, the boxes show distributions of estimated proportion changes of the eight cell types in autistic case samples compared to healthy control based on 100 bootstrapping of cells in Darmanis dataset.

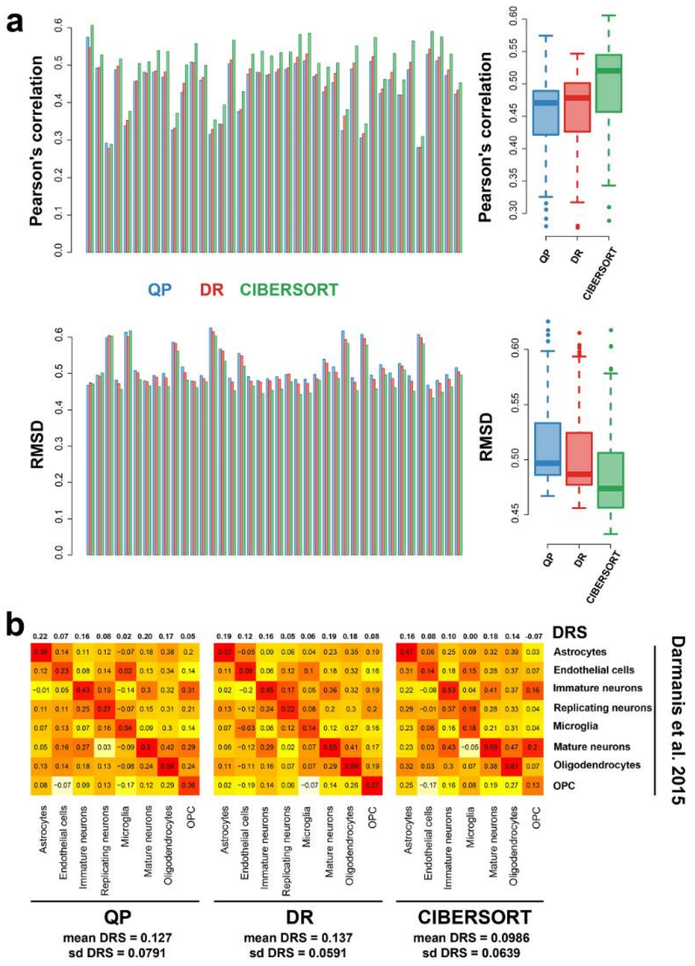

Supplementary Figure 4. Comparison of the three deconvolution algorithms: QP, DR and CIBERSORT. (a) Performance estimation using the similarity measurements, including the Pearson's correlation coefficient (PCC, upper) and root-mean-square deviation (RMSD, lower) between the observed bulk expression and the predicted bulk expression based on estimated cell type compositions. Colors of bars and boxes represent different algorithms. Each triple of bars in the left panel shows PCC and RMSD of each sample. Boxes in the right panel show PCC and RMSD across all samples. (b) Performance estimated based on the discrimination measurement, i.e. discriminating recovery score (DRS). The heatmaps show PCC between the re-estimated cell type transcriptome (columns) and the measured cell type transcriptome by Darmanis et al. 2015 [21] (rows). Each of the three heatmaps represents the results based on QP-based (left), DR-based (middle) and CIBERSORT-based (right) composition patterns, respectively. Numbers in the heatmaps show the PCC, with colors representing the relative PCCs in each column. The DRS of each cell type is shown above each column of the heatmaps, with the mean DRSs with the standard deviations shown at the bottom.

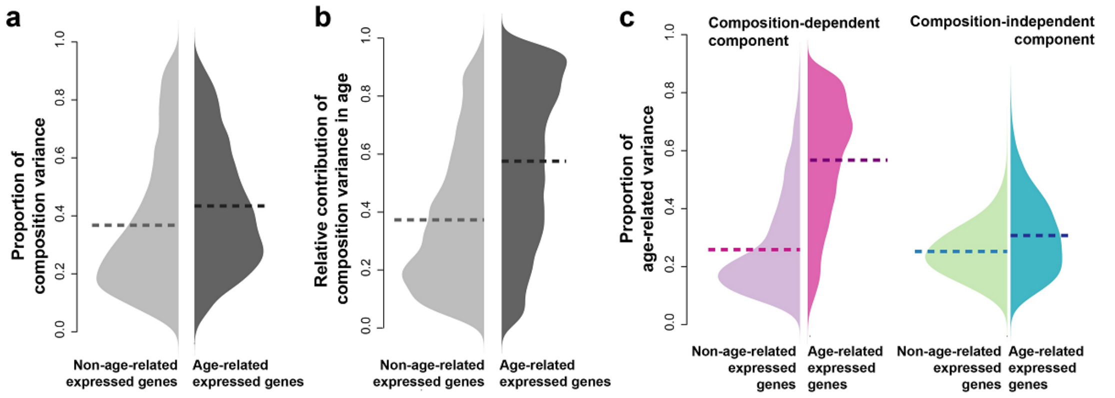

Supplementary Figure 5. The impact of cell type composition changes in age-related expressions, based on CIBERSORT-based composition patterns. (a) The proportions of gene expression variance explained by cell type composition (composition-related variance) in the human postnatal brain data set. Light grey – genes without age-related expression; dark grey – genes with age-related expression. The horizontal dash lines show the mean proportion of composition-related variance. (b) The relative contribution of the composition-dependent component to the variance explained by age (age-explained variance), measured as ratio of age-explained variance in the composition-dependent component to the sum of age-explained variance in both components. Light grey – genes without age-related expression; dark grey – genes with age-related expression. The horizontal dash lines show the mean proportion of composition-related variance. (c) The proportion of variance explained by ages (age-explained variance) in each of the two components of expression: pink – the composition-dependent component; green – the composition-independent component. The light colors represent the proportions of age-related variance in genes without age-related expression changes; the dark colors represent the proportions of age-related variance in genes with age-related expression changes. The horizontal dash lines show the means of age-related variance proportions.

Postnatal development

		DR				
		G1	G2	G3	Others	Total
CIBERSORT	G1	**400**	177	33	4	614
	G2	408	**5686**	37	246	6377
	G3	56	49	**256**	154	515
	Others	29	1351	236	**3855**	5471
	Total	893	7263	562	4259	

ASD alteration

		DR			
		Composition-independent	Composition-dependent only	Others	Total
CIBERSORT	Composition-independent	**229**	39	7	275
	Composition-dependent only	351	**6824**	647	7822
	Others	64	2937	**2934**	5935
	Total	644	9800	3588	

Supplementary Figure 6. DR and CIBERSORT based cell type composition patterns resulted in consistent classification of genes with composition-dependent and/or composition-independent component showing (a) age-related changes in postnatal human brains and (b) ASD alteration. The red shadows indicate significant overlap (hypergeometric test, P<0.0001).

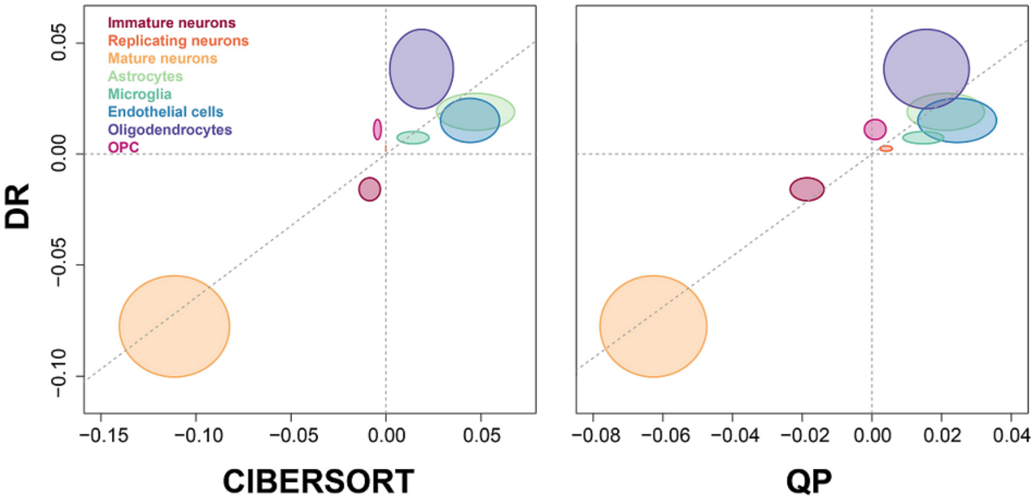

Supplementary Figure 7. Three deconvolution algorithms provide similar estimations of cell type composition alterations in ASD samples. Different ellipses represent the eight cell types. The center of each ellipse shows the average altered proportion of each cell type in ASD samples comparing to the healthy samples, based on the DR-based composition patterns (y-axis) or CIBERSORT (left panel) / QP (right panel) (x-axis) based composition patterns. The major and minor radius of each ellipse is proportional to the standard deviation of the alteration based on the corresponding algorithm.

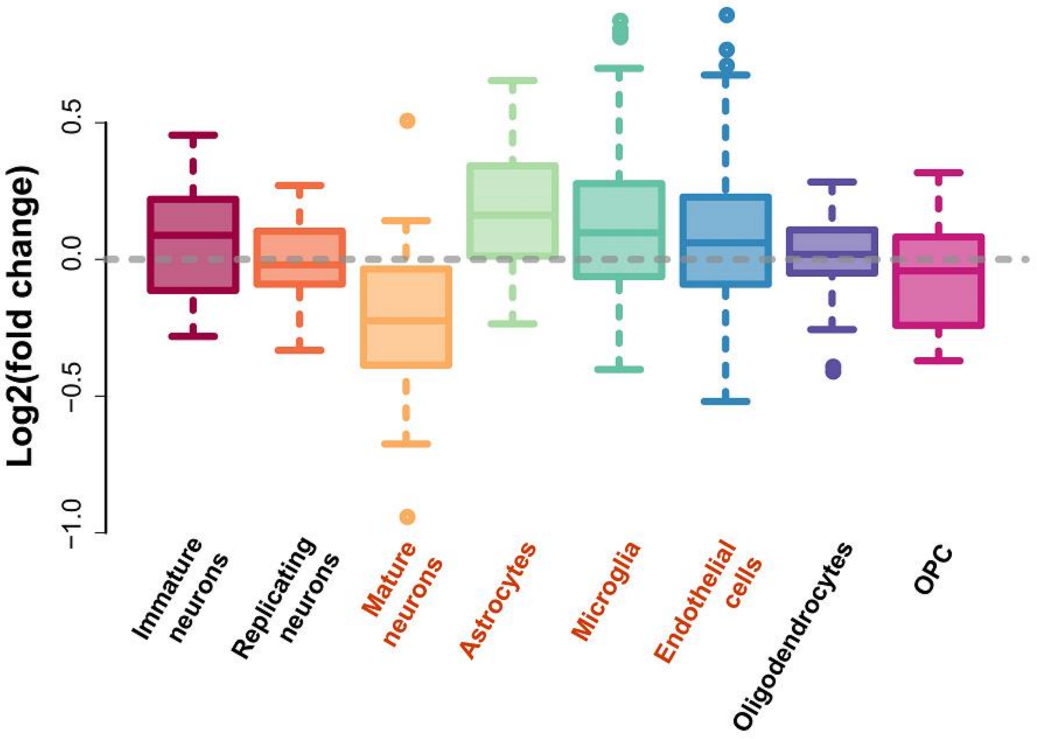

Supplementary Figure 8. Expression level alterations of cell type signature genes in autism samples, based on the Voineagu dataset. Each box shows the log2-transformed fold change of cell type signature genes for one cell type, between average expression levels in autistic samples and healthy samples. Labels of cell types whose signature genes show significant expression changes (Wilcoxon's test, BH corrected P<0.05) are marked in red.

Supplementary Table S1. Functional enrichment (DAVID) of each of the three groups of age-related changed genes, sorted in ascending order of Benjamini corrected *P* value. Only the functional terms with Benjamini corrected *P*<0.1 are shown.

G1 (Dependent+ Independent +)

**Category**	**Term**	**Count**	**%**	**PValue**	**List Total**	**Pop Hits**	**Pop Total**	**Fold Enrichment**	**Bonferroni**	**Benjamini**	**FDR**
UP_KEYWORDS	Cell membrane	161	19.24	5.10E-07	834	1582	11870	1.448	0.0002	0.0002	0.0007
GOTERM_BP_DIRECT	GO:0006414~translational elongation	21	2.51	1.66E-07	730	76	10388	3.932	0.0004	0.0004	0.0003
UP_KEYWORDS	Synapse	40	4.78	1.13E-05	834	269	11870	2.116	0.0047	0.0016	0.0158
UP_KEYWORDS	Cell junction	61	7.29	9.40E-06	834	483	11870	1.797	0.0040	0.0020	0.0132
GOTERM_CC_DIRECT	GO:0030425~dendrite	37	4.42	1.65E-05	770	245	10996	2.157	0.0071	0.0071	0.0232
GOTERM_CC_DIRECT	GO:0030054~cell junction	45	5.38	4.74E-05	770	339	10996	1.896	0.0204	0.0103	0.0669
GOTERM_BP_DIRECT	GO:0016259~selenocysteine metabolic process	17	2.03	2.02E-05	730	70	10388	3.456	0.0503	0.0128	0.0358
GOTERM_BP_DIRECT	GO:0006614~SRP-dependent cotranslational protein targeting to membrane	19	2.27	3.15E-05	730	88	10388	3.072	0.0772	0.0133	0.0556
GOTERM_BP_DIRECT	GO:0006413~translational initiation	22	2.63	1.60E-05	730	108	10388	2.899	0.0401	0.0135	0.0283
GOTERM_BP_DIRECT	GO:0006415~translational termination	17	2.03	1.12E-05	730	67	10388	3.611	0.0281	0.0142	0.0198
GOTERM_BP_DIRECT	GO:0000184~nuclear-transcribed mRNA catabolic process, nonsense-mediated decay	20	2.39	3.11E-05	730	96	10388	2.965	0.0762	0.0157	0.0549
UP_KEYWORDS	Calcium	60	7.17	1.87E-04	834	524	11870	1.630	0.0759	0.0195	0.2625
GOTERM_BP_DIRECT	GO:0019083~viral transcription	19	2.27	5.86E-05	730	92	10388	2.939	0.1389	0.0211	0.1035
GOTERM_BP_DIRECT	GO:0007268~synaptic transmission	38	4.54	8.00E-05	730	273	10388	1.981	0.1846	0.0252	0.1413

GOTERM_BP_DIRECT	GO:0006412~translation	32	3.82	1.59E-04	730	221	10388	2.060	0.3338	0.0398	0.2810
GOTERM_BP_DIRECT	GO:0001887~selenium compound metabolic process	18	2.15	1.47E-04	730	90	10388	2.846	0.3131	0.0409	0.2599
GOTERM_CC_DIRECT	GO:0022625~cytosolic large ribosomal subunit	12	1.43	3.76E-04	770	48	10996	3.570	0.1509	0.0531	0.5297
GOTERM_MF_DIRECT	GO:0004683~calmodulin-dependent protein kinase activity	8	0.96	7.75E-05	722	17	10423	6.794	0.0582	0.0582	0.1184
UP_KEYWORDS	Postsynaptic cell membrane	20	2.39	9.04E-04	834	123	11870	2.314	0.3172	0.0735	1.2631
GOTERM_CC_DIRECT	GO:0045211~postsynaptic membrane	23	2.75	8.67E-04	770	152	10996	2.161	0.3142	0.0900	1.2165
UP_KEYWORDS	Potassium transport	13	1.55	0.00145	834	64	11870	2.891	0.4589	0.0973	2.0251

G2 (Dependent+ Independent-)

Category	Term	Count	%	PValue	List Total	Pop Hits	Pop Total	Fold Enrichment	Bonferroni	Benjamini	FDR
UP_KEYWORDS	Zinc-finger	900	13.5849	4.43E-28	6594	1293	11870	1.2530	0.0000	0.0000	0.0000
UP_KEYWORDS	Nucleus	2277	34.3698	1.58E-22	6594	3667	11870	1.1178	0.0000	0.0000	0.0000
INTERPRO	IPR001909:Krueppel-associated box	225	3.39623	2.04E-23	6228	267	11196	1.5149	0.0000	0.0000	0.0000
SMART	SM00349:SM00349	225	3.39623	2.37E-22	3429	266	6011	1.4828	0.0000	0.0000	0.0000
UP_SEQ_FEATURE	domain:KRAB	214	3.23019	6.46E-22	6533	255	11760	1.5107	0.0000	0.0000	0.0000
UP_KEYWORDS	Zinc	1062	16.0302	6.39E-18	6594	1629	11870	1.1736	0.0000	0.0000	0.0000
UP_SEQ_FEATURE	zinc finger region:C2H2-type 9	232	3.50189	3.93E-18	6533	291	11760	1.4351	0.0000	0.0000	0.0000
INTERPRO	IPR015880:Zinc finger, C2H2-like	427	6.44528	6.69E-18	6228	590	11196	1.3010	0.0000	0.0000	0.0000
UP_SEQ_FEATURE	zinc finger region:C2H2-type 6	288	4.34717	2.48E-18	6533	374	11760	1.3862	0.0000	0.0000	0.0000
UP_SEQ_FEATURE	zinc finger region:C2H2-type 7	269	4.06038	3.73E-18	6533	346	11760	1.3995	0.0000	0.0000	0.0000

INTERPRO	IPR007087:Zinc finger, C2H2	410	6.18868	5.67E-18	6228	563	11196	1.3092	0.0000	0.0000	0.0000
INTERPRO	IPR013087:Zinc finger C2H2-type/integrase DNA-binding domain	361	5.44906	1.95E-17	6228	489	11196	1.3271	0.0000	0.0000	0.0000
UP_SEQ_FEATURE	zinc finger region:C2H2-type 10	211	3.18491	3.88E-17	6533	263	11760	1.4442	0.0000	0.0000	0.0000
UP_SEQ_FEATURE	zinc finger region:C2H2-type 3	337	5.08679	5.22E-17	6533	454	11760	1.3362	0.0000	0.0000	0.0000
SMART	SM00355:SM00355	427	6.44528	8.96E-16	3429	590	6011	1.2687	0.0000	0.0000	0.0000
UP_SEQ_FEATURE	zinc finger region:C2H2-type 8	250	3.77358	2.74E-16	6533	323	11760	1.3933	0.0000	0.0000	0.0000
UP_SEQ_FEATURE	zinc finger region:C2H2-type 5	309	4.66415	2.12E-16	6533	412	11760	1.3501	0.0000	0.0000	0.0000
GOTERM_MF_DIRECT	GO:0003677~DNA binding	672	10.1434	1.87E-16	5820	987	10423	1.2193	0.0000	0.0000	0.0000
UP_SEQ_FEATURE	zinc finger region:C2H2-type 4	317	4.78491	7.86E-16	6533	427	11760	1.3364	0.0000	0.0000	0.0000
UP_SEQ_FEATURE	zinc finger region:C2H2-type 2	323	4.87547	5.79E-15	6533	440	11760	1.3214	0.0000	0.0000	0.0000
UP_SEQ_FEATURE	zinc finger region:C2H2-type 11	186	2.80755	1.17E-14	6533	233	11760	1.4370	0.0000	0.0000	0.0000
GOTERM_CC_DIRECT	GO:0005634~nucleus	2152	32.483	1.47E-14	6088	3551	10996	1.0946	0.0000	0.0000	0.0000
GOTERM_BP_DIRECT	GO:0006351~transcription, DNA-templated	831	12.5434	2.71E-15	5744	1268	10388	1.1852	0.0000	0.0000	0.0000
UP_KEYWORDS	DNA-binding	817	12.3321	6.02E-13	6594	1257	11870	1.1700	0.0000	0.0000	0.0000
UP_SEQ_FEATURE	zinc finger region:C2H2-type 1	289	4.36226	2.10E-13	6533	394	11760	1.3204	0.0000	0.0000	0.0000
GOTERM_BP_DIRECT	GO:0006355~regulation of transcription, DNA-templated	585	8.83019	1.01E-13	5744	871	10388	1.2147	0.0000	0.0000	0.0000
UP_SEQ_FEATURE	zinc finger region:C2H2-type 12	161	2.43019	4.96E-13	6533	201	11760	1.4419	0.0000	0.0000	0.0000
UP_SEQ_FEATURE	zinc finger region:C2H2-type 13	132	1.99245	1.38E-12	6533	160	11760	1.4851	0.0000	0.0000	0.0000
UP_KEYWORDS	Transcription regulation	990	14.9434	1.46E-11	6594	1562	11870	1.1409	0.0000	0.0000	0.0000
UP_KEYWORDS	Transcription	1024	15.4566	1.23E-11	6594	1619	11870	1.1386	0.0000	0.0000	0.0000
UP_SEQ_FEATURE	zinc finger region:C2H2-type 14	106	1.6	1.05E-11	6533	125	11760	1.5265	0.0000	0.0000	0.0000
UP_KEYWORDS	Metal-binding	1517	22.8981	1.52E-09	6594	2494	11870	1.0949	0.0000	0.0000	0.0000

UP_SEQ_FEATURE	zinc finger region:C2H2-type 16	76	1.14717	8.16E-10	6533	87	11760	1.5725	0.0000	0.0000	0.0000
UP_KEYWORDS	Chromatin regulator	180	2.71698	2.32E-08	6594	247	11870	1.3118	0.0000	0.0000	0.0000
UP_SEQ_FEATURE	zinc finger region:C2H2-type 15	91	1.37358	4.92E-09	6533	110	11760	1.4892	0.0001	0.0000	0.0000
INTERPRO	IPR013083:Zinc finger, RING/FYVE/PHD-type	228	3.44151	1.95E-08	6228	322	11196	1.2729	0.0001	0.0000	0.0000
UP_KEYWORDS	Ubl conjugation pathway	338	5.10189	6.34E-07	6594	511	11870	1.1907	0.0004	0.0000	0.0009
GOTERM_BP_DIRECT	GO:0010467~gene expression	499	7.53208	5.21E-08	5744	773	10388	1.1675	0.0004	0.0001	0.0001
UP_KEYWORDS	Chromosome	171	2.58113	3.46E-06	6594	244	11870	1.2616	0.0022	0.0002	0.0051
GOTERM_CC_DIRECT	GO:0005654~nucleoplasm	1234	18.6264	5.16E-07	6088	2048	10996	1.0883	0.0006	0.0003	0.0008
UP_SEQ_FEATURE	zinc finger region:C2H2-type 17	61	0.92075	5.35E-07	6533	72	11760	1.5251	0.0060	0.0003	0.0011
INTERPRO	IPR001841:Zinc finger, RING-type	155	2.33962	5.02E-07	6228	214	11196	1.3021	0.0026	0.0004	0.0010
SMART	SM00184:SM00184	152	2.29434	2.69E-06	3429	209	6011	1.2749	0.0016	0.0005	0.0040
UP_KEYWORDS	RNA-binding	324	4.89057	9.92E-06	6594	498	11870	1.1712	0.0062	0.0006	0.0147
UP_SEQ_FEATURE	zinc finger region:C2H2-type 1; degenerate	72	1.08679	1.37E-06	6533	89	11760	1.4563	0.0153	0.0008	0.0028
GOTERM_MF_DIRECT	GO:0046872~metal ion binding	832	12.5585	8.87E-07	5820	1343	10423	1.1095	0.0021	0.0011	0.0016
GOTERM_MF_DIRECT	GO:0008270~zinc ion binding	502	7.57736	1.38E-06	5820	785	10423	1.1453	0.0033	0.0011	0.0024
GOTERM_BP_DIRECT	GO:0006996~organelle organization	175	2.64151	8.47E-07	5744	248	10388	1.2762	0.0064	0.0016	0.0017
KEGG_PATHWAY	hsa05016:Huntington's disease	97	1.46415	1.87E-05	2257	137	4284	1.3439	0.0055	0.0027	0.0248
KEGG_PATHWAY	hsa05012:Parkinson's disease	71	1.0717	9.65E-06	2257	94	4284	1.4337	0.0028	0.0028	0.0128
INTERPRO	IPR013164:Cadherin, N-terminal	39	0.58868	3.94E-06	6228	43	11196	1.6305	0.0205	0.0030	0.0075
UP_KEYWORDS	mRNA processing	171	2.58113	6.35E-05	6594	252	11870	1.2215	0.0389	0.0030	0.0943
UP_SEQ_FEATURE	zinc finger region:RING-type	105	1.58491	5.44E-06	6533	141	11760	1.3405	0.0595	0.0031	0.0112
UP_KEYWORDS	mRNA splicing	139	2.09811	5.95E-05	6594	200	11870	1.2511	0.0364	0.0031	0.0883

GOTERM_MF_DIRECT	GO:0004842~ubiquitin-protein transferase activity	159	2.4	5.32E-06	5820	225	10423	1.2656	0.0127	0.0032	0.0093
GOTERM_MF_DIRECT	GO:0016874~ligase activity	144	2.17358	7.37E-06	5820	202	10423	1.2767	0.0175	0.0035	0.0129
GOTERM_MF_DIRECT	GO:0044822~poly(A) RNA binding	582	8.78491	1.20E-05	5820	931	10423	1.1195	0.0283	0.0048	0.0210
KEGG_PATHWAY	hsa00190:Oxidative phosphorylation	61	0.92075	1.01E-04	2257	82	4284	1.4120	0.0292	0.0074	0.1343
UP_KEYWORDS	Ligase	200	3.01887	1.77E-04	6594	303	11870	1.1882	0.1044	0.0078	0.2620
UP_KEYWORDS	Cell cycle	327	4.93585	1.95E-04	6594	516	11870	1.1408	0.1144	0.0081	0.2887
UP_KEYWORDS	Helicase	85	1.28302	2.08E-04	6594	117	11870	1.3078	0.1216	0.0081	0.3082
KEGG_PATHWAY	hsa05010:Alzheimer's disease	84	1.26792	8.96E-05	2257	119	4284	1.3398	0.0260	0.0087	0.1193
UP_KEYWORDS	Mental retardation	158	2.38491	2.47E-04	6594	235	11870	1.2103	0.1430	0.0090	0.3665
UP_KEYWORDS	Ubl conjugation	788	11.8943	2.82E-04	6594	1311	11870	1.0820	0.1613	0.0097	0.4177
SMART	SM00249:SM00249	62	0.93585	6.76E-05	3429	78	6011	1.3934	0.0400	0.0102	0.0998
UP_KEYWORDS	Cell division	204	3.07925	3.34E-04	6594	312	11870	1.1770	0.1880	0.0109	0.4943
UP_KEYWORDS	RNA-mediated gene silencing	31	0.46792	4.19E-04	6594	36	11870	1.5501	0.2299	0.0130	0.6197
UP_KEYWORDS	Isopeptide bond	491	7.41132	4.86E-04	6594	801	11870	1.1034	0.2617	0.0137	0.7193
UP_KEYWORDS	Acetylation	1628	24.5736	4.79E-04	6594	2792	11870	1.0496	0.2582	0.0141	0.7082
INTERPRO	IPR001965:Zinc finger, PHD-type	62	0.93585	2.56E-05	6228	78	11196	1.4289	0.1261	0.0167	0.0490
INTERPRO	IPR019787:Zinc finger, PHD-finger	55	0.83019	3.40E-05	6228	68	11196	1.4540	0.1640	0.0197	0.0651
UP_KEYWORDS	Kelch repeat	40	0.60377	7.99E-04	6594	50	11870	1.4401	0.3927	0.0214	1.1796
SMART	SM00490:SM00490	69	1.04151	1.80E-04	3429	90	6011	1.3440	0.1034	0.0216	0.2663
UP_SEQ_FEATURE	domain:Helicase ATP-binding	70	1.0566	4.60E-05	6533	91	11760	1.3847	0.4051	0.0244	0.0950
INTERPRO	IPR001650:Helicase, C-terminal	70	1.0566	4.86E-05	6228	91	11196	1.3828	0.2254	0.0252	0.0929
GOTERM_BP_DIRECT	GO:0022904~respiratory electron transport chain	64	0.96604	2.00E-05	5744	81	10388	1.4289	0.1400	0.0297	0.0398
UP_KEYWORDS	Centromere	68	1.02642	0.00122	6594	94	11870	1.3022	0.5323	0.0312	1.7922

SMART	SM00487:SM00487	69	1.04151	3.18E-04	3429	91	6011	1.3292	0.1751	0.0316	0.4693
UP_SEQ_FEATURE	zinc finger region:C2H2-type 19	40	0.60377	6.73E-05	6533	47	11760	1.5320	0.5324	0.0340	0.1390
UP_KEYWORDS	Ribonucleoprotein	142	2.1434	1.60E-03	6594	215	11870	1.1889	0.6321	0.0377	2.3513
UP_KEYWORDS	Nucleotide-binding	786	11.8642	0.00159	6594	1322	11870	1.0703	0.6305	0.0390	2.3415
UP_SEQ_FEATURE	domain:Helicase C-terminal	68	1.02642	8.74E-05	6533	89	11760	1.3754	0.6271	0.0403	0.1804
UP_KEYWORDS	DNA damage	173	2.61132	0.00179	6594	267	11870	1.1664	0.6731	0.0406	2.6252
INTERPRO	IPR014001:Helicase, superfamily 1/2, ATP-binding domain	70	1.0566	8.89E-05	6228	92	11196	1.3678	0.3737	0.0416	0.1700
UP_SEQ_FEATURE	zinc finger region:C2H2-type 18	46	0.69434	8.73E-05	6533	56	11760	1.4786	0.6267	0.0419	0.1802
GOTERM_BP_DIRECT	GO:0000278~mitotic cell cycle	228	3.44151	4.59E-05	5744	346	10388	1.1917	0.2922	0.0423	0.0910
GOTERM_BP_DIRECT	GO:0016567~protein ubiquitination	182	2.74717	4.43E-05	5744	270	10388	1.2191	0.2835	0.0465	0.0878
GOTERM_MF_DIRECT	GO:0003723~RNA binding	209	3.15472	1.52E-04	5820	316	10423	1.1845	0.3054	0.0507	0.2664
GOTERM_BP_DIRECT	GO:0044237~cellular metabolic process	83	1.25283	6.51E-05	5744	112	10388	1.3402	0.3875	0.0530	0.1291
UP_SEQ_FEATURE	zinc finger region:C2H2-type 2; degenerate	43	0.64906	1.21E-04	6533	52	11760	1.4885	0.7442	0.0531	0.2492
GOTERM_BP_DIRECT	GO:0008380~RNA splicing	134	2.02264	4.41E-05	5744	192	10388	1.2622	0.2825	0.0538	0.0874
UP_KEYWORDS	DNA replication	58	0.87547	0.00279	6594	80	11870	1.3051	0.8254	0.0584	4.0678
UP_KEYWORDS	DNA repair	145	2.18868	2.75E-03	6594	222	11870	1.1758	0.8203	0.0595	4.0027
UP_KEYWORDS	Mitosis	137	2.06792	0.00298	6594	209	11870	1.1800	0.8445	0.0602	4.3319
UP_KEYWORDS	Spliceosome	71	1.0717	0.00313	6594	101	11870	1.2654	0.8589	0.0612	4.5526
GOTERM_MF_DIRECT	GO:0000166~nucleotide binding	155	2.33962	2.38E-04	5820	229	10423	1.2122	0.4351	0.0689	0.4171
GOTERM_MF_DIRECT	GO:0061630~ubiquitin protein ligase activity	97	1.46415	2.68E-04	5820	136	10423	1.2773	0.4745	0.0690	0.4699
UP_KEYWORDS	Nucleosome core	23	0.34717	0.00448	6594	27	11870	1.5334	0.9393	0.0838	6.4509
UP_KEYWORDS	Respiratory chain	31	0.46792	0.00469	6594	39	11870	1.4309	0.9469	0.0851	6.7476

UP_KEYWORDS	Neurogenesis	125	1.88679	4.98E-03	6594	191	11870	1.1781	0.9556	0.0876	7.1450
UP_KEYWORDS	ATP-binding	609	9.19245	0.00531	6594	1024	11870	1.0706	0.9639	0.0906	7.6015
GOTERM_MF_DIRECT	GO:0003700~transcription factor activity, sequence-specific DNA binding	364	5.49434	4.05E-04	5820	580	10423	1.1239	0.6219	0.0927	0.7093
INTERPRO	IPR012677:Nucleotide-binding, alpha-beta plait	130	1.96226	2.33E-04	6228	189	11196	1.2365	0.7060	0.0970	0.4442

G3 (Dependent- Independent+)

Category	Term	Count	%	PValue	List Total	Pop Hits	Pop Total	Fold Enrichment	Bonferroni	Benjamini	FDR
UP_KEYWORDS	Secreted	80	15.444	1.45E-12	517	790	11870	2.3250	0.0000	0.0000	0.0000
UP_KEYWORDS	Signal	130	25.0965	4.92E-11	517	1706	11870	1.7495	0.0000	0.0000	0.0000
UP_KEYWORDS	Disulfide bond	106	20.4633	4.69E-09	517	1385	11870	1.7572	0.0000	0.0000	0.0000
UP_SEQ_FEATURE	signal peptide	123	23.7452	4.62E-10	516	1623	11760	1.7272	0.0000	0.0000	0.0000
GOTERM_CC_DIRECT	GO:0070062~extracellular exosome	133	25.6757	5.21E-09	489	1856	10996	1.6114	0.0000	0.0000	0.0000
GOTERM_CC_DIRECT	GO:0005576~extracellular region	57	11.0039	2.50E-08	489	580	10996	2.2099	0.0000	0.0000	0.0000
GOTERM_CC_DIRECT	GO:0005615~extracellular space	60	11.583	6.71E-08	489	644	10996	2.0950	0.0000	0.0000	0.0001
UP_SEQ_FEATURE	disulfide bond	90	17.3745	2.55E-08	516	1134	11760	1.8088	0.0000	0.0000	0.0000
UP_SEQ_FEATURE	topological domain:Extracellular	94	18.1467	9.18E-08	516	1237	11760	1.7319	0.0001	0.0000	0.0002
INTERPRO	IPR006703:AIG1	7	1.35135	1.69E-07	487	8	11196	20.1160	0.0002	0.0002	0.0003
UP_KEYWORDS	Glycoprotein	137	26.4479	2.73E-06	517	2179	11870	1.4435	0.0010	0.0003	0.0038
GOTERM_CC_DIRECT	GO:0009897~external side of plasma membrane	18	3.4749	3.34E-06	489	105	10996	3.8549	0.0012	0.0003	0.0046
GOTERM_CC_DIRECT	GO:0031012~extracellular matrix	18	3.4749	5.64E-06	489	109	10996	3.7134	0.0019	0.0004	0.0077

UP_SEQ_FEATURE	topological domain:Cytoplasmic	112	21.6216	6.57E-06	516	1705	11760	1.4971	0.0095	0.0024	0.0109
UP_KEYWORDS	Cell adhesion	32	6.17761	4.21E-05	517	329	11870	2.2331	0.0153	0.0031	0.0579
UP_KEYWORDS	Cell membrane	101	19.4981	5.32E-05	517	1582	11870	1.4658	0.0193	0.0032	0.0733
GOTERM_CC_DIRECT	GO:0005887~integral component of plasma membrane	54	10.4247	5.75E-05	489	690	10996	1.7598	0.0196	0.0033	0.0785
UP_SEQ_FEATURE	glycosylation site:N-linked (GlcNAc...)	126	24.3243	1.32E-05	516	2007	11760	1.4308	0.0190	0.0038	0.0219
UP_KEYWORDS	Transmembrane	161	31.0811	7.72E-05	517	2823	11870	1.3094	0.0279	0.0040	0.1062
UP_KEYWORDS	Fatty acid metabolism	14	2.7027	1.03E-04	517	88	11870	3.6526	0.0369	0.0042	0.1413
UP_KEYWORDS	Transmembrane helix	160	30.888	9.58E-05	517	2814	11870	1.3054	0.0344	0.0044	0.1317
UP_KEYWORDS	Cytokine	12	2.3166	1.52E-04	517	68	11870	4.0517	0.0542	0.0056	0.2092
UP_KEYWORDS	Lipid metabolism	26	5.01931	2.84E-04	517	269	11870	2.2191	0.0988	0.0094	0.3908
KEGG_PATHWAY	hsa05144:Malaria	9	1.73745	4.61E-05	225	27	4284	6.3467	0.0107	0.0107	0.0592
UP_KEYWORDS	Lysosome	20	3.861	4.40E-04	517	185	11870	2.4821	0.1489	0.0123	0.6046
UP_KEYWORDS	Oxidoreductase	32	6.17761	4.10E-04	517	373	11870	1.9697	0.1394	0.0124	0.5633
GOTERM_BP_DIRECT	GO:0006955~immune response	20	3.861	1.39E-05	458	142	10388	3.1945	0.0300	0.0151	0.0242
GOTERM_CC_DIRECT	GO:0005886~plasma membrane	126	24.3243	4.03E-04	489	2139	10996	1.3246	0.1298	0.0197	0.5486
GOTERM_BP_DIRECT	GO:0007155~cell adhesion	30	5.79151	9.42E-06	458	273	10388	2.4924	0.0204	0.0204	0.0164
SMART	SM00181:SM00181	17	3.28185	1.15E-04	244	137	6011	3.0569	0.0210	0.0210	0.1420
BIOCARTA	h_granulocytesPathway:Adhesion and Diapedesis of Granulocytes	6	1.1583	1.75E-04	67	11	1169	9.5170	0.0213	0.0213	0.2018
UP_KEYWORDS	EGF-like domain	16	3.0888	0.00112	517	140	11870	2.6239	0.3354	0.0288	1.5251
KEGG_PATHWAY	hsa04060:Cytokine-cytokine receptor interaction	15	2.89575	3.52E-04	225	95	4284	3.0063	0.0788	0.0402	0.4515
UP_KEYWORDS	Immunity	20	3.861	0.00264	517	215	11870	2.1358	0.6197	0.0624	3.5718
UP_SEQ_FEATURE	transmembrane region	153	29.5367	3.02E-04	516	2709	11760	1.2872	0.3554	0.0706	0.4993

INTERPRO	IPR000742:Epidermal growth factor-like domain	18	3.4749	1.64E-04	487	144	11196	2.8737	0.1396	0.0724	0.2563
UP_KEYWORDS	Deafness	14	2.7027	0.00349	517	127	11870	2.5310	0.7223	0.0770	4.7055
UP_KEYWORDS	Lectin	10	1.9305	0.00422	517	73	11870	3.1451	0.7870	0.0869	5.6510
SMART	SM00060:SM00060	14	2.7027	0.00151	244	125	6011	2.7591	0.2440	0.0890	1.8538

Supplementary Table S2. Functional enrichment (DAVID) of genes with altered expressions in ASD in their composition-independent components, sorted in ascending order of Benjamini corrected *P* value. Only the functional terms with Benjamini corrected *P*<0.1 are shown.

Category	Term	Count	%	PValue	List Total	Pop Hits	Pop Total	Fold Enrichment	Bonferroni	Benjamini	FDR
SP_PIR_KEYWORDS	membrane	279	44.71	4.09E-13	618	4176	13242	1.4316	0.0000	0.0000	0.0000
SP_PIR_KEYWORDS	transmembrane	216	34.62	8.86E-11	618	3133	13242	1.4773	0.0000	0.0000	0.0000
UP_SEQ_FEATURE	transmembrane region	216	34.62	4.46E-11	617	3115	13233	1.4872	0.0000	0.0000	0.0000
UP_SEQ_FEATURE	topological domain:Cytoplasmic	152	24.36	1.65E-10	617	1986	13233	1.6415	0.0000	0.0000	0.0000
SP_PIR_KEYWORDS	glycoprotein	174	27.88	5.00E-09	618	2480	13242	1.5034	0.0000	0.0000	0.0000
SP_PIR_KEYWORDS	cell junction	38	6.09	6.99E-08	618	302	13242	2.6961	0.0000	0.0000	0.0001
UP_SEQ_FEATURE	glycosylation site:N-linked (GlcNAc...)	163	26.12	5.05E-08	617	2357	13233	1.4832	0.0001	0.0000	0.0001
UP_SEQ_FEATURE	topological domain:Extracellular	113	18.11	4.91E-08	617	1463	13233	1.6566	0.0001	0.0000	0.0001
GOTERM_CC_FAT	GO:0031224~intrinsic to membrane	240	38.46	1.02E-07	470	3478	8845	1.2986	0.0000	0.0000	0.0001
GOTERM_CC_FAT	GO:0045202~synapse	38	6.09	4.71E-07	470	289	8845	2.4745	0.0002	0.0001	0.0007
GOTERM_CC_FAT	GO:0016021~integral to membrane	229	36.70	6.72E-07	470	3347	8845	1.2876	0.0003	0.0001	0.0009
GOTERM_CC_FAT	GO:0005886~plasma membrane	173	27.72	3.45E-07	470	2337	8845	1.3931	0.0001	0.0001	0.0005
GOTERM_CC_FAT	GO:0044459~plasma membrane part	120	19.23	2.87E-06	470	1522	8845	1.4838	0.0011	0.0002	0.0040
SP_PIR_KEYWORDS	synapse	24	3.85	4.03E-06	618	170	13242	3.0250	0.0017	0.0003	0.0057
GOTERM_CC_FAT	GO:0044456~synapse part	28	4.49	5.31E-06	470	198	8845	2.6613	0.0021	0.0003	0.0074
GOTERM_CC_FAT	GO:0016023~cytoplasmic membrane-bounded vesicle	46	7.37	7.01E-06	470	428	8845	2.0226	0.0027	0.0004	0.0097
SP_PIR_KEYWORDS	signal	127	20.35	5.58E-06	618	1860	13242	1.4630	0.0024	0.0004	0.0079
GOTERM_CC_FAT	GO:0031988~membrane-bounded vesicle	47	7.53	8.51E-06	470	445	8845	1.9876	0.0033	0.0004	0.0118
SP_PIR_KEYWORDS	ion transport	39	6.25	1.71E-05	618	394	13242	2.1210	0.0072	0.0010	0.0240
UP_SEQ_FEATURE	signal peptide	127	20.35	5.42E-06	617	1860	13233	1.4644	0.0092	0.0018	0.0091
GOTERM_CC_FAT	GO:0030659~cytoplasmic vesicle membrane	18	2.88	4.83E-05	470	108	8845	3.1365	0.0186	0.0021	0.0670

GOTERM_CC_FAT	GO:0012506~vesicle membrane	19	3.04	5.62E-05	470	120	8845	2.9797	0.0216	0.0022	0.0781
GOTERM_CC_FAT	GO:0031410~cytoplasmic vesicle	48	7.69	9.59E-05	470	504	8845	1.7923	0.0365	0.0034	0.1331
SP_PIR_KEYWORDS	Immunoglobulin domain	28	4.49	6.74E-05	618	257	13242	2.3345	0.0282	0.0036	0.0948
GOTERM_CC_FAT	GO:0031982~vesicle	49	7.85	1.58E-04	470	529	8845	1.7432	0.0593	0.0044	0.2186
GOTERM_CC_FAT	GO:0008021~synaptic vesicle	13	2.08	1.54E-04	470	66	8845	3.7068	0.0580	0.0046	0.2134
SP_PIR_KEYWORDS	voltage-gated channel	15	2.40	1.08E-04	618	94	13242	3.4192	0.0450	0.0046	0.1525
SP_PIR_KEYWORDS	disulfide bond	99	15.87	9.91E-05	618	1456	13242	1.4569	0.0411	0.0047	0.1393
SP_PIR_KEYWORDS	transport	90	14.42	1.22E-04	618	1301	13242	1.4823	0.0502	0.0047	0.1708
GOTERM_CC_FAT	GO:0005887~integral to plasma membrane	65	10.42	1.47E-04	470	767	8845	1.5948	0.0553	0.0047	0.2033
SP_PIR_KEYWORDS	ionic channel	23	3.69	1.73E-04	618	201	13242	2.4519	0.0708	0.0061	0.2434
GOTERM_CC_FAT	GO:0030136~clathrin-coated vesicle	17	2.72	2.57E-04	470	112	8845	2.8565	0.0951	0.0066	0.3569
GOTERM_CC_FAT	GO:0031226~intrinsic to plasma membrane	65	10.42	2.82E-04	470	785	8845	1.5583	0.1037	0.0068	0.3912
GOTERM_CC_FAT	GO:0030135~coated vesicle	19	3.04	3.45E-04	470	138	8845	2.5910	0.1253	0.0078	0.4781
GOTERM_CC_FAT	GO:0030054~cell junction	38	6.09	4.51E-04	470	393	8845	1.8197	0.1605	0.0097	0.6243
GOTERM_CC_FAT	GO:0044433~cytoplasmic vesicle part	19	3.04	4.91E-04	470	142	8845	2.5181	0.1734	0.0100	0.6794
GOTERM_CC_FAT	GO:0030662~coated vesicle membrane	12	1.92	5.49E-04	470	65	8845	3.4743	0.1919	0.0106	0.7597
GOTERM_CC_FAT	GO:0030665~clathrin coated vesicle membrane	10	1.60	5.83E-04	470	46	8845	4.0911	0.2025	0.0107	0.8067
GOTERM_BP_FAT	GO:0006812~cation transport	40	6.41	1.76E-05	466	397	9616	2.0791	0.0389	0.0131	0.0307
GOTERM_BP_FAT	GO:0019226~transmission of nerve impulse	31	4.97	2.97E-05	466	278	9616	2.3010	0.0647	0.0133	0.0517
GOTERM_BP_FAT	GO:0000904~cell morphogenesis involved in differentiation	25	4.01	2.44E-05	466	196	9616	2.6320	0.0535	0.0137	0.0425
GOTERM_BP_FAT	GO:0006811~ion transport	50	8.01	1.31E-05	466	540	9616	1.9107	0.0291	0.0146	0.0228
GOTERM_BP_FAT	GO:0007268~synaptic transmission	27	4.33	5.33E-05	466	232	9616	2.4015	0.1132	0.0149	0.0928
GOTERM_BP_FAT	GO:0048667~cell morphogenesis involved in neuron differentiation	22	3.53	5.28E-05	466	167	9616	2.7184	0.1122	0.0169	0.0920
UP_SEQ_FEATURE	disulfide bond	96	15.38	6.43E-05	617	1387	13233	1.4845	0.1039	0.0181	0.1085

GOTERM_BP_FAT	GO:0030001~metal ion transport	36	5.77	8.22E-06	466	328	9616	2.2648	0.0184	0.0184	0.0143
GOTERM_BP_FAT	GO:0006816~calcium ion transport	16	2.56	7.48E-05	466	99	9616	3.3350	0.1553	0.0186	0.1304
GOTERM_BP_FAT	GO:0031175~neuron projection development	25	4.01	5.11E-05	466	205	9616	2.5165	0.1087	0.0190	0.0890
GOTERM_BP_FAT	GO:0048812~neuron projection morphogenesis	22	3.53	8.87E-05	466	173	9616	2.6241	0.1813	0.0198	0.1545
SP_PIR_KEYWORDS	cell membrane	80	12.82	6.21E-04	618	1182	13242	1.4502	0.2315	0.0201	0.8697
GOTERM_BP_FAT	GO:0030182~neuron differentiation	32	5.13	1.34E-04	466	316	9616	2.0896	0.2609	0.0249	0.2334
GOTERM_BP_FAT	GO:0048666~neuron development	28	4.49	1.25E-04	466	258	9616	2.2395	0.2457	0.0253	0.2177
SP_PIR_KEYWORDS	cell adhesion	28	4.49	8.70E-04	618	301	13242	1.9932	0.3086	0.0260	1.2167
GOTERM_BP_FAT	GO:0007409~axonogenesis	20	3.21	1.67E-04	466	155	9616	2.6626	0.3144	0.0286	0.2914
GOTERM_BP_FAT	GO:0050877~neurological system process	48	7.69	2.16E-04	466	571	9616	1.7347	0.3861	0.0342	0.3765
SP_PIR_KEYWORDS	calcium	42	6.73	0.00129	618	538	13242	1.6728	0.4225	0.0359	1.8048
GOTERM_BP_FAT	GO:0015674~di-, tri-valent inorganic cation transport	17	2.72	3.01E-04	466	124	9616	2.8290	0.4924	0.0442	0.5229
GOTERM_BP_FAT	GO:0016337~cell-cell adhesion	21	3.37	4.60E-04	466	181	9616	2.3941	0.6458	0.0628	0.7992
GOTERM_CC_FAT	GO:0012505~endomembrane system	52	8.33	0.00368	470	659	8845	1.4850	0.7608	0.0630	4.9906
GOTERM_CC_FAT	GO:0030672~synaptic vesicle membrane	6	0.96	0.00411	470	21	8845	5.3769	0.7981	0.0672	5.5650
GOTERM_BP_FAT	GO:0032990~cell part morphogenesis	23	3.69	5.47E-04	466	211	9616	2.2493	0.7090	0.0700	0.9499
GOTERM_MF_FAT	GO:0022843~voltage-gated cation channel activity	15	2.40	5.11E-04	441	106	9174	2.9438	0.3119	0.0720	0.7726
SP_PIR_KEYWORDS	transmembrane protein	31	4.97	0.00291	618	376	13242	1.7666	0.7095	0.0744	4.0184
GOTERM_BP_FAT	GO:0000902~cell morphogenesis	28	4.49	6.27E-04	466	285	9616	2.0273	0.7569	0.0756	1.0873
GOTERM_BP_FAT	GO:0048858~cell projection morphogenesis	22	3.53	6.63E-04	466	200	9616	2.2699	0.7757	0.0757	1.1491
GOTERM_BP_FAT	GO:0007157~heterophilic cell adhesion	6	0.96	7.25E-04	466	16	9616	7.7382	0.8051	0.0785	1.2565
GOTERM_BP_FAT	GO:0007155~cell adhesion	41	6.57	8.41E-04	466	493	9616	1.7161	0.8501	0.0792	1.4565
GOTERM_BP_FAT	GO:0022610~biological adhesion	41	6.57	8.86E-04	466	494	9616	1.7126	0.8644	0.0799	1.5332
GOTERM_BP_FAT	GO:0007267~cell-cell signaling	35	5.61	7.85E-04	466	396	9616	1.8238	0.8298	0.0809	1.3598
GOTERM_BP_FAT	GO:0007156~homophilic cell adhesion	13	2.08	8.31E-04	466	86	9616	3.1193	0.8467	0.0817	1.4396

GOTERM_MF_FAT	GO:0031420~alkali metal ion binding	19	3.04	5.08E-04	441	157	9174	2.5175	0.3102	0.0887	0.7676
GOTERM_MF_FAT	GO:0005509~calcium ion binding	50	8.01	3.83E-04	441	624	9174	1.6669	0.2440	0.0890	0.5787
GOTERM_MF_FAT	GO:0046873~metal ion transmembrane transporter activity	25	4.01	1.36E-04	441	220	9174	2.3639	0.0949	0.0949	0.2067
INTERPRO	IPR002473:Small chemokine, C-X-C/Interleukin 8	5	0.80	2.89E-04	555	8	11800	13.2883	0.2611	0.0959	0.4586

Supplementary Dataset S1. The CIBERSORT-selected markers used in CIBERSORT and QP algorithms, and the cell type signature genes of the eight main cell types in human brains used in DR algorithms.

Supplementary Dataset S2. ANCOVA results indicating age-related changes in postnatal human brains in composition-dependent or composition-independent components.

Supplementary Dataset S3. ANCOVA results indicating ASD alteration in composition-dependent or composition-independent components.
